# Microbial and metabolomic profiling of the upper respiratory tract in children with asthma

**DOI:** 10.3389/fmicb.2026.1672589

**Published:** 2026-02-17

**Authors:** Lina Xu, Qianjun Wan, Quying Yang, Wenxin Shen, Yinfang Dai, Huiquan Sun, Li Huang, Meijuan Wang, Wujun Jiang, Chuangli Hao

**Affiliations:** 1 Department of Respiratory Medicine, Children’s Hospital of Soochow University, Suzhou, China; 2 Emergency Department, Maternal and Child Health Hospital of Hubei Province, Wuhan, China

**Keywords:** asthma, children, metabolomics, microbiota-metabolome interaction, upper respiratory tract microbiome

## Abstract

**Background:**

This study aimed to investigate characteristic changes in the upper respiratory tract (URT) microbiome and metabolome in children with asthma and explore their associations with lung function.

**Methods:**

Children with asthma aged 6 years and above admitted to the Children’s Hospital of Soochow University from December 2022 to December 2023 comprised the study group. Age-matched healthy children undergoing physical examinations in the Department of Child Health were recruited as controls. Throat swabs were collected for microbiome detection using 16S rDNA sequencing and metabolomics analysis using liquid chromatography-mass spectrometry (LC–MS).

**Results:**

(1) Significant differences in alpha and beta diversity were observed among the control group (H), chronic persistent asthma group (CA), and acute exacerbation group (AA). In both CA and AA groups, FVC% predicted (FVC%/Pred) and FEV1% predicted (FEV1%/Pred) were negatively correlated with URT microbiota abundance. *Actinobacillus* abundance was positively correlated with FEV1%/Pred, FEV1/FVC, FEF25%/Pred, FEF50%/Pred, and FEF75%/Pred. (2) Metabolite differences between CA and AA groups were analyzed, and the top 5 differential metabolites were evaluated for their accuracy as asthma assessment biomarkers. L-carnitine showed an AUC > 0.9, with a sensitivity of 85.7% and specificity of 85%. Other differential metabolites, including monoisobutyl phthalate, 4-hexyl-2,5-dimethyloxazole, and dibutyl phthalate, correlated with several lung function indices. The most relevant differential metabolic pathways included arginine biosynthesis, alanine-aspartate–glutamate metabolism, central carbon metabolism in cancer, and D-amino acid metabolism.

**Conclusion:**

The URT microbiota in asthmatic children exhibits alterations in composition, structure, and diversity, with lower diversity in acute asthma compared to chronic persistent asthma. At the genus level, some microbiota (*Actinobacillus*, *Fusobacterium*) were correlated with FEV1%/Pred, FEV1/FVC, FEF25%/Pred, FEF50%/Pred, FEF75%/Pred. The differential metabolite L-carnitine may be a potential biomarker for asthma assessment.

## Introduction

Bronchial asthma, a heterogeneous respiratory disease characterized by chronic airway inflammation and reversible airflow obstruction, has shown an alarming increase in pediatric prevalence over the past three decades (Yang et al., 2024). The pathogenesis of asthma involves complex interactions among genetic susceptibility, environmental exposures, and microbial dysbiosis; however, its underlying mechanisms remain incompletely understood.

The upper respiratory tract (URT) microbiome, as an essential part of the mucosal ecosystem, plays a vital role in airway development, immune modulation, and protection against pathogens (Man et al., 2017; Lupu et al., 2023). Recent studies highlight its importance in asthma pathogenesis, yet most investigations have focused mainly on microbial composition (e.g., diversity, abundance) (Park et al., 2014), rather than functional metabolic activity. To complement taxonomic data, metabolomics, a comprehensive analysis of small-molecule metabolites, offers direct insight into host–microbe interactions and microbial function (Cobos-Uribe and Rebuli, 2023).

School-age children represent a critical population for URT microbiome studies, as their immune systems are still developing and they are typically exposed to specific environments such as schools, which together shape characteristic patterns of microbial colonization in the upper airway (Resztak et al., 2023; Rutter et al., 2025). Although the gut microbiota and adult respiratory microbiota have been studied extensively (Kullberg et al., 2021), the interplay between the URT microbiome and the metabolome in pediatric asthma remains largely unexplored, especially regarding its association with lung function.

Therefore, this study employed 16S rRNA sequencing and LC–MS–based metabolomics to comprehensively profile the URT microbial and metabolic features among children with chronic persistent asthma (CA), acute exacerbation (AA), and healthy controls (H), aiming to elucidate microbe–metabolite interactions associated with disease severity and airway dysfunction.

## Methods

### Patients and clinical information

School-age children (≥6 years) diagnosed with bronchial asthma according to the Global Initiative for Asthma (GINA 2022^1^) guidelines were enrolled from the Children’s Hospital of Soochow University between December 2022 and December 2023. Asthmatic participants were stratified into chronic persistent (CA) and acute exacerbation (AA) groups based on GINA criteria. Age-matched healthy controls (H) were recruited from the Child Health Department during routine physical examinations.

The inclusion criteria were: (1) Age ≥ 6 years; (2) Confirmed asthma diagnosis per GINA guidelines; (3) Absence of systemic infectious diseases; (4) Ability to cooperate with throat swab collection.

The exclusion criteria were: (1) Respiratory infection symptoms or signs within 7 days; (2) Recent contact (≤7 days) with individuals with respiratory infections; (3) Positive etiological tests results during clinical visits; (4) Antibiotic or other treatment within 2 weeks; (5) Comorbidities (cardiac, pulmonary, hepatic, renal, thyroid, neurological, autoimmune, or malignant diseases); (6) ICU admission for asthma exacerbation within the past year.

Demographic data (gender, age, medical history), medication use, treatment duration, and lung function parameters were recorded. The study protocol was approved by the Institutional Review Board of Soochow University (approval no. 2018KS009), and written informed consent was obtained from all guardians.

Throat swab collection was performed as follows: (1) Participants fasted for ≥1 h prior to sampling; (2) Using sterile swabs, the bilateral palatine arches and posterior pharynx were firmly swabbed 2–3 times, with avoidance of the tongue or buccal mucosa; (3) Swabs were immediately placed in cryotubes and flash-frozen in liquid nitrogen.

**Due to the low-biomass nature of pediatric throat swab specimens, untargeted metabolomic profiling was feasible only for a subset of samples.

### Lung function tests

Spirometry was performed using a Jaeger Masterscreen Pneumo system (CareFusion, Germany) according to ATS/ERS guidelines. Participants abstained from caffeine-containing beverages on the test day. Measurements were obtained in triplicate with the participants seated, wearing a nose clip, and using a disposable mouthpiece. Acceptability required a variation of <5% or 100 mL variation between the two highest forced vital capacity (FVC) maneuvers. The following parameters were recorded: Forced Expiratory Volume in 1 s (FEV₁), Forced Vital Capacity (FVC), FEV₁/FVC ratio, Forced Expiratory Flow at 25–75% of predicted value (FEF25–75%pred), Peak Expiratory Flow (PEF), and Maximal Expiratory Flow at 50% of FVC (MEF50%). Percent predicted values (e.g., FEV₁%pred, FVC%pred, FEF25–75%pred) were calculated based on reference equations for Chinese children. All tests were performed by trained technicians, and quality control followed ATS/ERS standards.

### Microbiome detection (16S rDNA sequencing) and bioinformatic processing

Total microbial DNA from throat swabs was extracted using the CTAB method following standardized procedures. DNA integrity and purity were assessed by agarose gel electrophoresis and NanoDrop spectrophotometry (Thermo Fisher Scientific), and DNA concentrations were quantified using the Qubit dsDNA High-Sensitivity Assay (Thermo Fisher Scientific). The V3–V4 hypervariable region of the 16S rRNA gene was amplified using primers 341F (5′-CCTAYGGGRBGCASCAG-3′) and 806R (5′-GGACTACNNGGGTATCTAAT-3′). PCR products were purified with AMPure XP beads, quantified using Qubit, pooled in equimolar concentrations, and then sequenced on an Illumina NovaSeq 6000 platform (2 × 250 bp paired-end).

Raw paired-end reads were demultiplexed and processed using QIIME2 (v2021.11). Adapter and primer sequences were removed using Cutadapt. Quality filtering, denoising, and amplicon sequence variant (ASV) inference were performed using the DADA2 plugin. Based on per-base quality profiles, the first 10 low-quality bases were trimmed from both forward and reverse reads (trimLeft = 10/10), and forward and reverse reads were truncated at 240 bp and 200 bp, respectively (truncLen = 240/200). Reads with more than two expected errors (maxEE = 2), Phred quality scores below 20, or remaining length <200 bp after trimming were removed. Samples were retained only if ≥80% of bases achieved Q30. Chimeric sequences were identified and removed using the consensus-based removeBimeraDenovo method. Singleton ASVs and extremely low-abundance features were excluded according to standard practice to minimize sequencing noise.

Taxonomic classification of ASVs was conducted using the QIIME2 naïve Bayes classifier trained on the SILVA 138 reference database trimmed to the V3–V4 region (confidence threshold: 70%). Non-bacterial and unclassified ASVs were excluded before downstream analysis. Alpha diversity indices (Chao1, Shannon) were calculated from rarefied ASV tables. Beta diversity was computed using Bray–Curtis dissimilarity and weighted and unweighted UniFrac distance matrices and visualized by principal coordinates analysis (PCoA). Global community differences were assessed using PERMANOVA with 999 permutations.

Differentially enriched taxa were identified using LEfSe with an LDA score threshold of >3.5. To account for the compositional nature of sequencing data, differential analysis was further validated using ANCOM-BC with Benjamini–Hochberg false discovery rate correction (FDR < 0.05). Only taxa consistently identified across both methods were considered robust (Takai and Horikoshi, 2000; Walters et al., 2016).

### Untargeted metabolomics by LC–MS

Throat-swab extracts from a subset of participants (CA *n* = 28; AA *n* = 20; H *n* = 27) were subjected to untargeted metabolomic profiling. Within this metabolomics-eligible sample pool, a stratified random sampling strategy was applied according to clinical groups (CA, AA, and H) to minimize potential selection bias. Sample selection was conducted prior to data analysis and was independent of microbiome profiles or clinical outcomes. After thawing on ice, 20 μL of each sample was extracted with 120 μL of pre-cooled 50% methanol, vortexed for 1 min, incubated for 10 min at room temperature, and precipitated at −20 °C overnight. Following centrifugation (4,000 × g, 20 min, 4 °C), supernatants were collected and stored at −80 °C until analysis. A pooled QC sample was prepared by mixing aliquots of all samples.

Metabolite separation was performed on an ACQUITY UPLC T3 column (100 × 2.1 mm, 1.8 μm) with a mobile-phase gradient of water (5 mM ammonium acetate + 5 mM acetic acid) and acetonitrile at a flow rate of 0.3 mL/min. Mass spectrometry was conducted on a SCIEX TripleTOF 6600 in positive and negative ESI modes (m/z 60–1,200), using information-dependent acquisition with a dynamic exclusion of 4 s. QC samples were injected every 10 runs.

Raw data were processed using XCMS for peak detection, retention-time alignment, and deconvolution (centWave; ppm = 30; peakwidth = 5–25 s; snthresh = 6). Batch effects and signal drift were corrected using QC-based robust LOESS signal correction. Features with >50% missing values in QC samples or >80% missing values within any group were excluded. Metabolite annotation was performed by accurate mass and MS/MS spectral matching against HMDB (v5.0) and KEGG compound databases, using a mass tolerance of ±10 ppm and MS/MS similarity ≥0.8.

**Figure 1:**
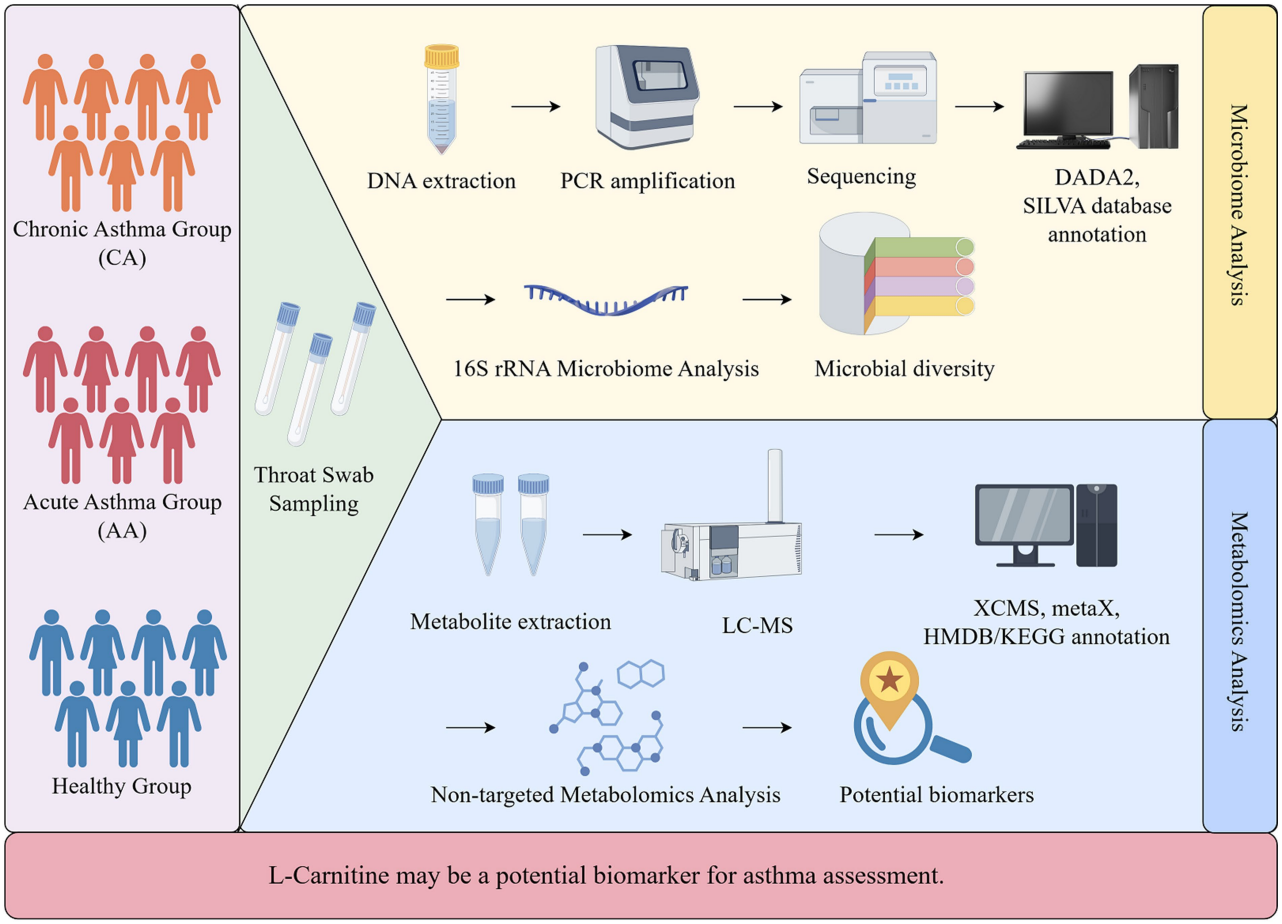
Study design and flow chart.

After probabilistic quotient normalization and log transformation, PCA and PLS-DA were used to evaluate metabolic separation among groups. Differential metabolites were identified using VIP > 1.0, fold change >1.5 or <0.67, and Benjamini–Hochberg–adjusted *p* < 0.05. Enriched metabolic pathways were analyzed using the KEGG database (release 2023) via hypergeometric testing with FDR correction (Figure 1). PLS-DA and VI*p* values for metabolomic analysis were calculated using the ropls or equivalent multivariate analysis framework.

### Statistical analysis

Clinical data were analyzed using SPSS 27.0 (IBM, United States). Continuous variables with non-normal distribution are presented as median (IQR) and compared using the Kruskal–Wallis test. Categorical variables are presented as counts (%) and compared using the χ^2^ test or Fisher’s exact test, as appropriate. A two-sided P value <0.05 was considered statistically significant. Multiple testing correction was performed using the Benjamini–Hochberg false discovery rate (FDR) where applicable.

## Results

### Characteristic analysis of upper respiratory microbiome

#### Study population

A total of 99 participants were included in the microbiome analysis: 78 with CA, 21 with AA, and 27 with H. No significant differences were observed in gender, age, or BMI across groups (*p* > 0.05, Supplementary Table S1). Lung function indices were significantly lower in the AA group than in the CA group (*p* < 0.01; Supplementary Table S2).

#### Microbial community composition

Analysis of the top 30 most abundant species revealed distinct compositional profiles at the phylum and genus levels across groups (Supplementary Figure S2). At the phylum level (Supplementary Figure S2A), *Firmicutes*, *Proteobacteria*, *Bacteroidota*, *Fusobacteriota*, and *Actinobacteriota* were the dominant phyla in all groups. Notably, the proportion of *Firmicutes* was significantly higher in the AA group (46.33%) compared to the H group (41.34%) (Kruskal-Wallis *p* < 0.05). At the genus level (Supplementary Figure S2E), *Streptococcus*, *Neisseria*, *Veillonella*, and *Prevotella* predominated. While the relative abundance of *Streptococcus* was similar across groups, the abundance of *Veillonella* was significantly higher in AA (14.74%) than in CA (8.84%) and H (7.31%; *p* < 0.05).

#### Alpha diversity analysis

**Figure 2:**
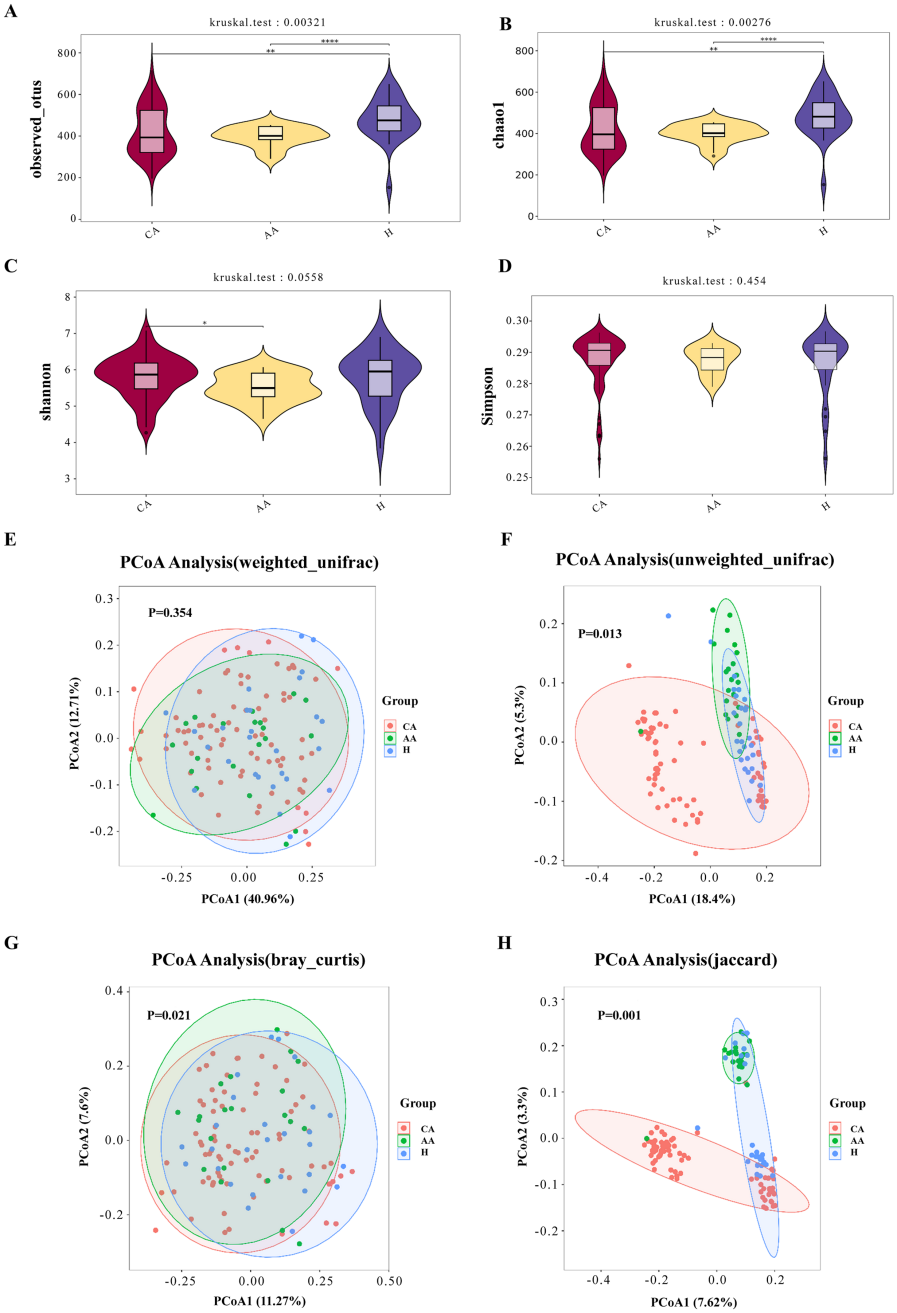
**(A)** Observed OTUs index among the CA, AA, and H groups. **(B)** Chao1 index among the CA, AA, and H groups. **(C)** Shannon index among the CA, AA, and H groups. **(D)** Simpson index among the CA, AA, and H groups. **(E)** PCoA based on weighted UniFrac distance of upper respiratory tract microbiota among the CA, AA, and H groups. **(F)** PCoA based on unweighted UniFrac distance of upper respiratory tract microbiota among the CA, AA, and H groups. **(G)** PCoA based on Bray–Curtis distance of upper respiratory tract microbiota among the CA, AA, and H groups. **(H)** PCoA based on Jaccard distance of upper respiratory tract microbiota among the CA, AA, and H groups. The *P*-values obtained from the rank sum test for all groups in the figure are shown above, where the significant differences between two groups are represented by *indicating *p* <0.05, **indicating *p* <0.01, and ****indicating *p*<0.001.

Analysis of α-diversity indices revealed significant differences (Figures 2A–D). Both Observed OTUs (richness) and Chao1 (estimated richness) were significantly lower in the CA and AA groups compared to the H group (*p* < 0.05; Figures 2A,B). Observed OTUs (mean ± SD): H, 480.67 ± 101.51; CA, 397.33 ± 47.26; AA, 419.69 ± 116.24. Chao1: H, 483.87 ± 101.89; CA, 421.45 ± 116.98; AA, 399.09 ± 47.35. While the Shannon (diversity) and Simpson (dominance) indices did not differ significantly among all three groups (*p* > 0.05; Figures 2C,D), a statistically significant reduction in the Shannon index was observed in the AA group (5.49 ± 0.41) compared to the CA group (5.79 ± 0.59) (*p* < 0.05; Figure 2C).

#### Beta diversity analysis

Using four distance metrics—weighted UniFrac, unweighted UniFrac, Bray-Curtis, and Jaccard—we compared sample distributions among the control group, chronic persistent asthma group, and acute exacerbation group. Additionally, Anosim (Analysis of similarities) was applied for similarity analysis. Significant intergroup differences (*p* < 0.05) were detected using unweighted UniFrac, Bray-Curtis, and Jaccard distances, but not using weighted UniFrac (*p* > 0.05; Figure 2).

#### Differential species analysis

**Figure 3:**
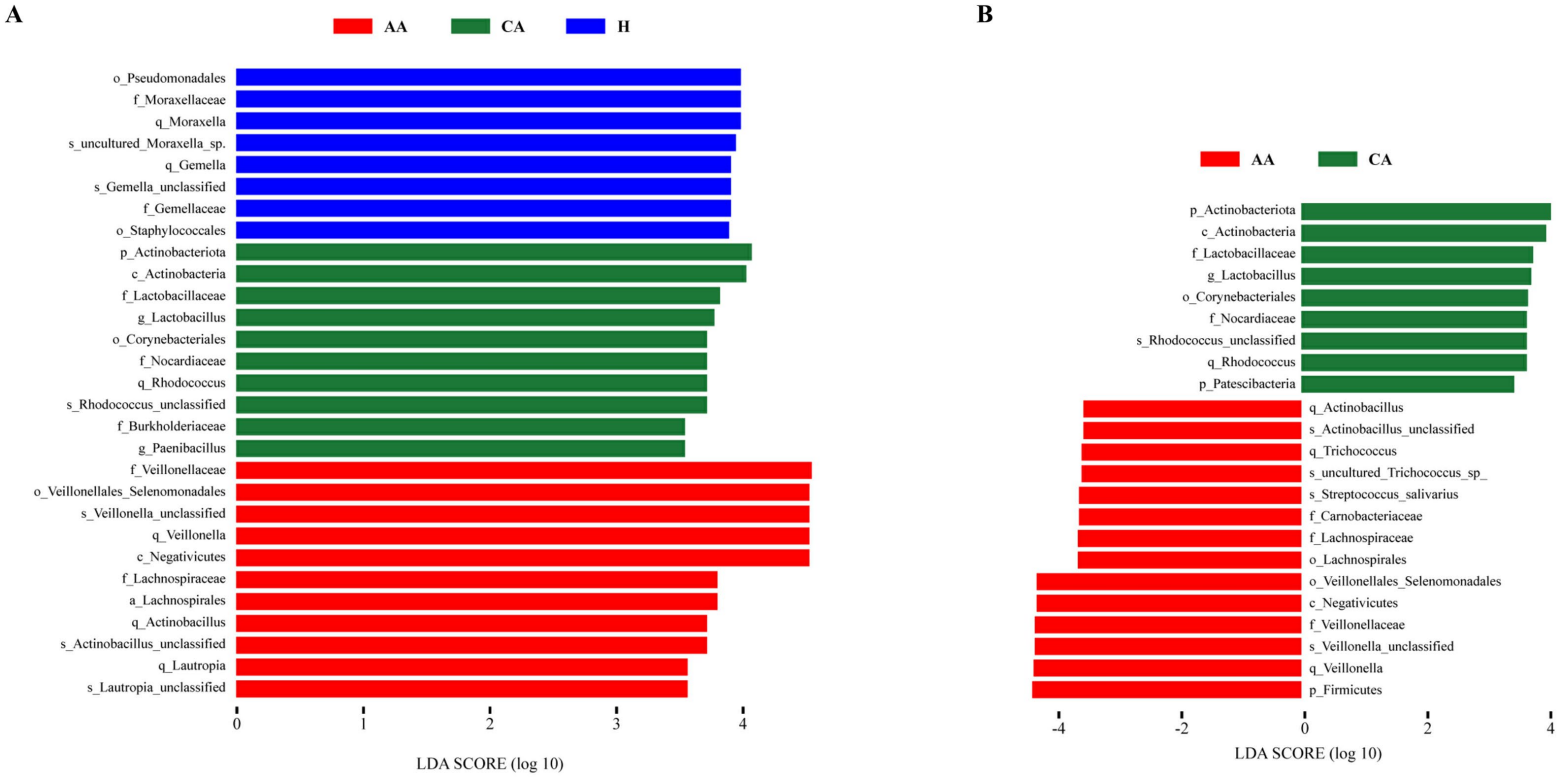
Panel **A** shows the LEfSe analysis of differential taxa among the AA, CA, and H groups, and Panel **B** shows the LEfSe analysis of differential taxa between the CA and AA groups.

LEfSe analysis identified taxa significantly enriched in each group (LDA score > 3.5; Figures 3A,B). Comparing all three groups (Figure 3A), the H group exhibited higher abundances of *Pseudomonadales*, *Moraxellaceae*, *Moraxella*, unclassified *Moraxella*, and *Gemella*. The CA group was enriched in *Actinobacteriota*, *Actinobacteria*, *Lactobacillaceae*, *Lactobacillus*, and *Corynebacteriales*. The AA group was characterized by higher abundances of *Veillonellaceae*, *Veillonellales-Selenomonadales*, unclassified *Veillonella*, *Veillonella*, and *Negativicutes*. Direct comparison between CA and AA groups (Figure 3B) confirmed the significant enrichment of *Firmicutes*, *Veillonella*, unclassified *Veillonella*, *Veillonellaceae*, and *Negativicutes* in AA, alongside depletion of *Actinobacteriota*, *Actinobacteria*, *Lactobacillaceae*, and *Lactobacillus*.

#### Integration of microbiome data with clinical parameters

**Figure 4:**
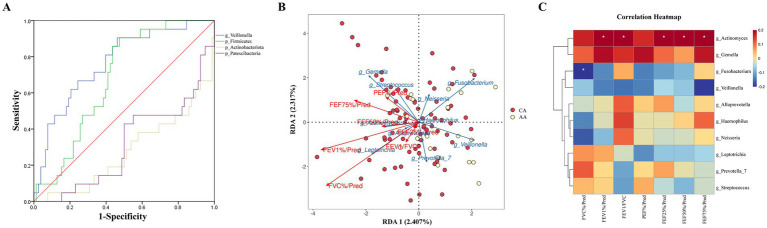
Associations between upper respiratory tract microbiota and lung function. **(A)** ROC curve of upper respiratory tract microbiota with significant differences in species at the phylum and genus levels and abundance in the top 10. **(B)** RDA analysis chart of upper respiratory tract microbiota and lung function test results in the top 10 levels of average abundance in CA and AA groups. **(C)** Spearman analysis chart of upper respiratory tract microbiota and lung function test results in the top 10 levels of average abundance in the chronic duration group and acute exacerbation group of asthma.

Using LEfSe to analyze species differences between AAand CAgroups, we selected the top 10 most abundant URT microbiota with significant differences at the phylum and genus levels to establish an asthma severity assessment model and plotted the ROC curve. *Veillonella* exhibited the highest diagnostic accuracy (AUC = 0.766; Figure 4A). Cross-validated ROC analysis confirmed *Veillonella* as a robust biomarker for asthma exacerbations (AUC = 0.76, 95% CI: 0.68–0.84).

Through species analysis, we selected the top 10 most abundant URT microbiota at the genus level in the CA and AA groups and conducted RDA analysis with lung function test results: RDA1 explained 2.41% of the variance, and RDA2 explained 2.32%. The envfit test showed that FVC%/Pred and FEV1%/Pred were negatively correlated with the abundance of URT microbiota (RDA1 = −0.88, *P* < 0.01; RDA1 = −0.96, *P* < 0.01), while other indicators showed no statistical association with URT microbiota (Figure 4B).

Likewise, species analysis of the top 10 most abundant URT microbiota at the genus level in the CA and AA groups was performed, followed by Spearman correlation analysis with lung function test results: *Actinobacillus* abundance was positively correlated with FEV1%/Pred, FEV1/FVC, FEF25%/Pred, FEF50%/Pred, and FEF75%/Pred (*r* = 0.21, *p* < 0.05; *r* = 0.21, *p* < 0.05; *r* = 0.21, *p* < 0.05; *r* = 0.24, *p* < 0.05; *r* = 0.22, *p* < 0.05); *Fusobacterium* abundance was negatively correlated with FVC%/Pred (*r* = −0.24, *p* < 0.05); no statistically significant correlations were observed between other URT genera and lung function indicators (Figure 4C).

### Metabolomics characteristic analysis

#### Clinical characteristics

The study enrolled 48 participants: 28 in CA group (median age: 10 years; male/female ratio: 16/12), 20 in AA group (median age: 9 years; male/female ratio: 11/9), and 27 H groups (median age: 9 years; male/female ratio: 16/11). No significant differences were observed in gender, age, BMI, or living environment across groups (*p* > 0.05; Supplementary Table S3). Lung function indices were significantly lower in the AA group compared to the CA group (*P* < 0.05; Supplementary Table S4).

#### Metabolomics profile analysis

**Figure 5:**
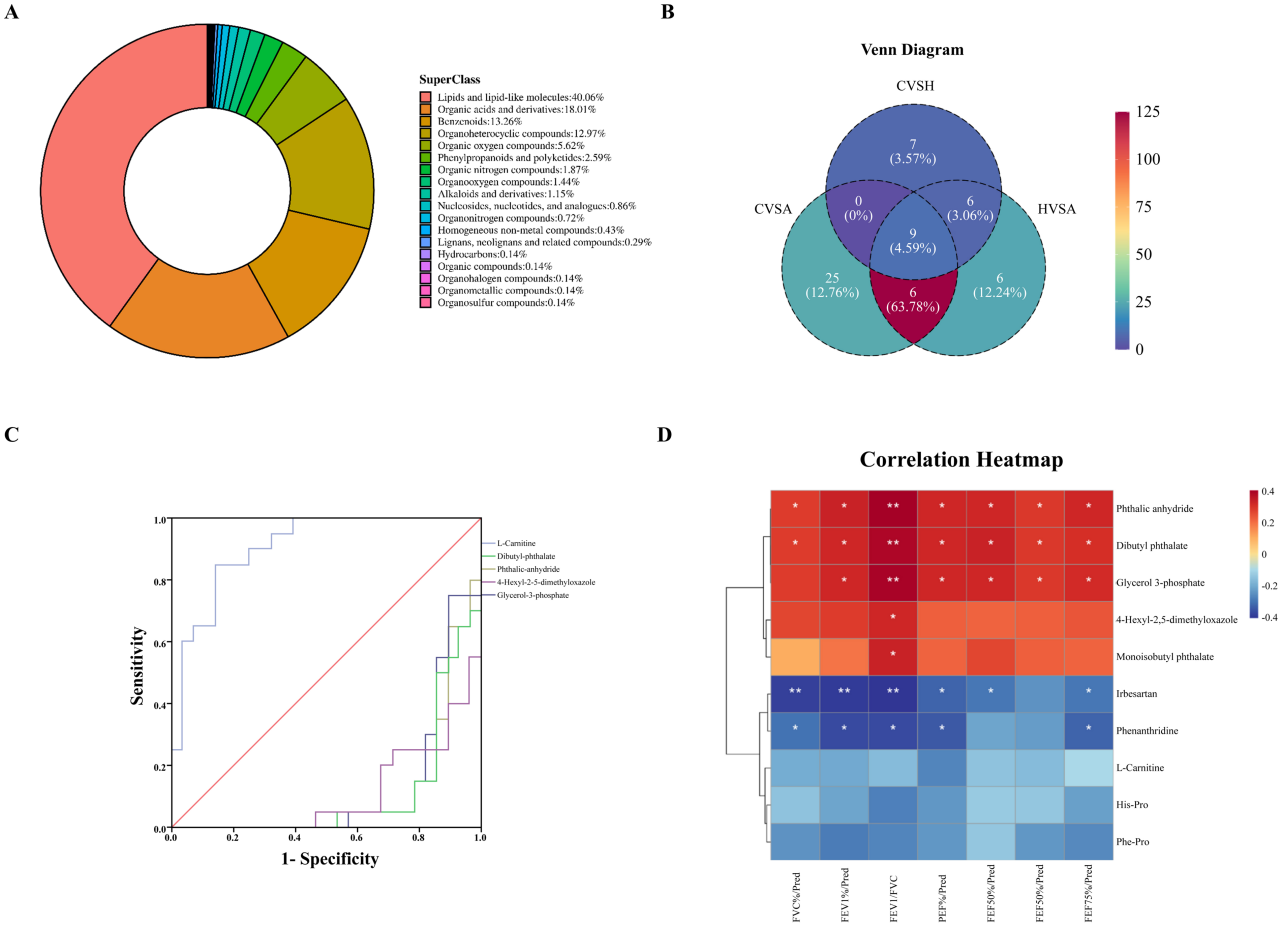
Metabolite profiles and clinical relevance in asthma. **(A)** Metabolite classification proportion chart. **(B)** Venn plot of differential metabolites among comparison groups. **(C)** ROC curve of the top 5 metabolites with differences between CA and AA group. **(D)** Spearman analysis chart of the top 10 differential metabolites and lung function test results in the chronic duration group and acute exacerbation group of asthma.

Untargeted metabolomics identified 803 metabolites (372 in positive ion mode; 431 in negative ion mode), 694 (86.4%) of which were annotated using the HMDB. Lipid and lipid-like molecules constituted the largest category (40.06%, 278 metabolites), followed by organic acids and derivatives (18.01%, 125 metabolites) and benzene-related compounds (13.26%, 92 metabolites) (Figure 5A).

### Screening of differential metabolites and analysis of lung function results

Differential Abundance: Venn analysis of differential metabolites (*p* < 0.05, |log₂FC| ≥ 0.26, VIP ≥ 1.0) identified 164 differentiating H group vs. AA group, 22 for H group vs. CA group, and 159 for CA group vs. AA group, with 9 shared across all comparisons (Figure 5B; Supplementary Table S6). Among the top 5 differential metabolites, L-carnitine exhibited the highest diagnostic power for asthma assessment (AUC = 0.91, 95% CI: 0.85–0.97; sensitivity 85.7%, specificity 85%; Figure 5C).

The top 10 differential metabolites (Table 1) in the CA and AA groups of asthma were selected for Spearman analysis with lung function test results, and an analysis chart was plotted. The results are shown below (Figure 5D): Dibutyl phthalate: Strongest correlation with FEV1/FVC (*r* = 0.39, *p* < 0.01) and significant associations with six other parameters (FVC%/Pred, FEV1%/Pred, PEF%/Pred, FEF25%/Pred, FEF50%/Pred, FEF75%/Pred; *r* = 0.28–0.33, all *p* < 0.05). Glycerol 3-phosphate: Positively correlated with FEV1%/Pred (r = 0.32, *p* = 0.03) and FEV1/FVC (*r* = 0.39, *p* < 0.01). Phthalic anhydride: Highest correlation with FEV1/FVC (*r* = 0.40, *p* < 0.01) across seven lung function indices. Negative correlations: Irbesartan showed strong inverse associations with FEV1%/Pred (*r* = −0.40, *p* < 0.01) and FEV1/FVC (*r* = −0.40, *p* < 0.01). Phenanthridine: Negatively correlated with FEV1%/Pred (*r* = −0.37, *p* = 0.01) and PEF%/Pred (*r* = −0.34, *p* = 0.02) (Figure 5D).

**Table 1:** The top 10 metabolites with differences were identified in the CA and AA groups.

Metabolite name	SuperClass	FC	Regulation	*P*	VIP
L-carnitine	Organic nitrogen compounds	0.23	Down	<0.001	1.41
Dibutyl phthalate	Benzenoids	11.05	Up	<0.001	2.84
Phthalic anhydride	–	12.37	Up	<0.001	3.02
4-Hexyl-2,5-dimethyloxazole	Organ heterocyclic compounds	6.46	Up	<0.001	1.59
Glycerol 3-phosphate	Lipids and lipid-like molecules	11.92	Up	<0.001	3.01
Phenanthridine	–	0.29	Down	<0.001	1.31
Monoisobutyl phthalate	Benzenoids	8.14	Up	<0.001	2.27
Phe-Pro	Organic acids and derivatives	0.30	Down	<0.001	1.12
Irbesartan	Benzenoids	0.42	Down	<0.001	1.17
His-Pro	Organic acids and derivatives	0.11	Down	<0.001	1.77

SuperClass: Metabolic superclass information from HMDB (Human Metabolome Database); FC: Fold change (expression difference); VIP: Variable importance in projection (predictive variable importance value).

### Analysis of pathways for differential metabolite enrichment

**Figure 6:**
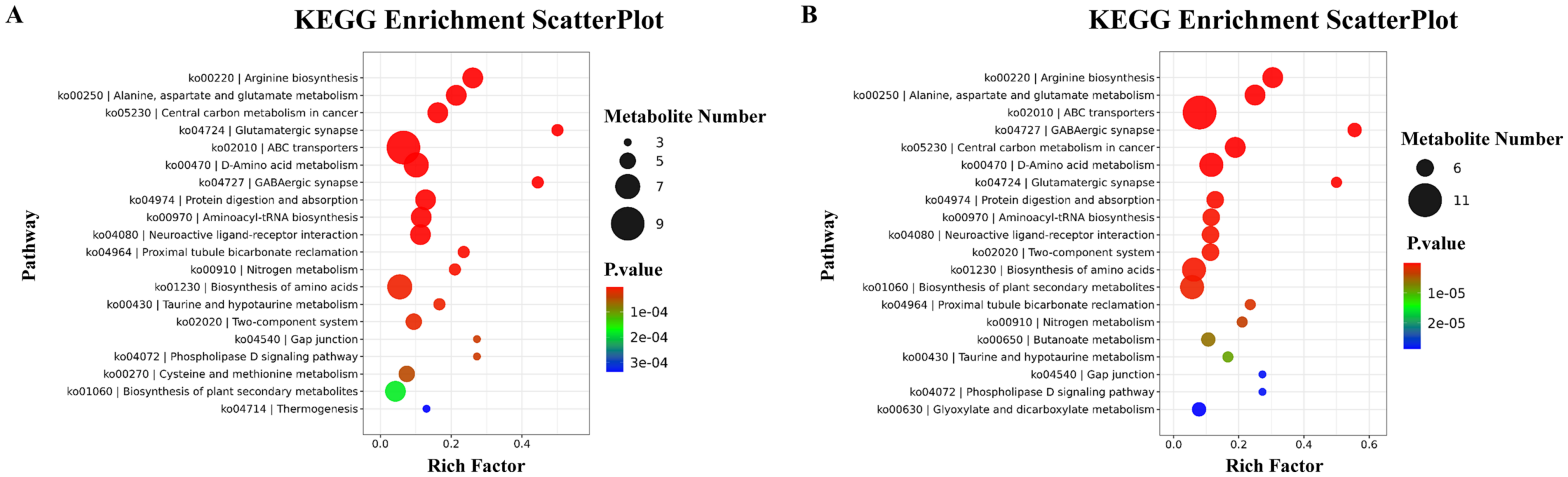
KEGG enrichment pathway analysis bubble chart. **(A)** KEGG pathway enrichment analysis of differential metabolites between the AA group and H group. **(B)** KEGG pathway enrichment analysis of differential metabolites between the CA group and AA group.

KEGG pathway enrichment analysis highlighted arginine biosynthesis (*p* = 3.1 × 10^−9^) and alanine-aspartate–glutamate metabolism (*p* = 1.1 × 10^−^8) as the most significantly altered pathways in AA vs. H (Figure 6A). Between CA and AA, arginine metabolism remained the core pathway (*p* = 2.6 × 10^−11^, Figure 6B), suggesting its persistent role in asthma progression.

## Discussion

Asthma is a heterogeneous respiratory disease characterized by chronic airway inflammation and reversible airflow obstruction (Hammad and Lambrecht, 2021; Kuruvilla et al., 2019). Despite extensive research, the functional interplay between the respiratory microbiota and host metabolism in pediatric asthma remains incompletely understood. Our study employs a dual-omics approach to characterize the upper respiratory microbiome and metabolome in school-age children across the CA, AA, and H groups.

### URT microbiome alterations: reduced diversity, shifting composition, and functional correlations

In this study of the oropharyngeal microbiome in asthma, we observed distinct ecological collapse in acute asthma, evidenced by reduced microbial richness (Observed OTUs, Chao1) and destabilized community structure (decreased Shannon index in AA vs. CA), which is consistent with previous studies (Park et al., 2014; Galeana-Cadena et al., 2023). Crucially, we observed a statistically significant decrease in the Shannon diversity index in the AA group compared to CA group (*p* < 0.05), suggesting that the bacterial community structure is more unstable during the AA phase. The excessive proliferation of dominant bacteria (e.g., *Veillonella*) may outcompete symbiotic bacteria for resources, thereby exacerbating ecological imbalance (Mejias and Ramilo, 2020). Beta diversity analysis further confirmed distinct microbial community compositions among the groups, with the AA microbiota showing the least similarity to both CA and H, underscoring significant restructuring during acute episodes. Multiple studies have reported differences in the lung and gut microbiomes between individuals with established asthma and healthy subjects, with the former exhibiting lower bacterial diversity (Zheng et al., 2022; Valverde-Molina and García-Marcos, 2023).

LEfSe analysis identified *Veillonella* as significantly enriched in AA group and correlated with exacerbation risk (AUC = 0.76), whereas *Lactobacillus* was depleted in the CA group. Our combined interpretation reveals critical functional associations between specific microbial shifts and metabolic dysregulation. *Veillonella* spp. are known to produce short-chain fatty acids (SCFAs), including propionate (Scheiman et al., 2019; Mashima et al., 2021). Although SCFAs can exert immunomodulatory effects, elevated propionate in the context of airway inflammation has been linked to the activation of the NLRP3 inflammasome, promoting IL-1β release and potentially skewing immune responses toward a Th17 phenotype—associated with neutrophilic inflammation and steroid resistance (Wang et al., 2024). This pro-inflammatory milieu is consistent with the heightened inflammation and poorer lung function observed in our AA group. The depletion of *Lactobacillus* in the chronic phase may indicate a loss of beneficial commensals with known immunomodulatory properties, such as promoting regulatory T-cell (Treg) responses and maintaining epithelial barrier integrity—potentially contributing to a dysregulated pro-inflammatory/anti-inflammatory balance in AA (Shen and Wong, 2016; Zhang et al., 2025).

Building on the observed microbial dysbiosis, we identified specific genus-level associations between URT taxa and lung function parameters, directly linking URT taxa to functional impairment: *Actinobacillus* abundance was positively correlated with FEV1%/Pred, FEV1/FVC, and small airway indices (FEF25-75%/Pred), whereas *Fusobacterium* abundance was negatively correlated with FVC%/Pred. Although the precise functional roles of these genera within the pediatric URT asthmatic niche require further investigation, these correlations directly link specific microbial signatures to the degree of airway obstruction.

### Metabolic dysregulation: L-carnitine depletion and arginine pathway centrality

To explore the functional significance of these microbial changes, we further characterized the metabolomic profiles. Most strikingly, L-carnitine emerged as a highly discriminatory metabolite, being significantly depleted in the AA group compared to the CA group, with exceptional diagnostic performance (AUC = 0.91). L-carnitine is essential for mitochondrial fatty acid β-oxidation (FAO), a crucial energy-generating process, and also plays a role in modulating inflammation and oxidative stress (Shumakova et al., 2022). Reduced L-carnitine levels may reflect impaired mitochondrial energetics and a metabolic shift toward glycolysis, a phenomenon commonly observed in inflammatory states, which could contribute to airway inflammation and functional impairment during acute asthma exacerbations. Intriguingly, the concurrent enrichment of *Veillonella* and depletion of L-carnitine raises the hypothesis that *Veillonella* overgrowth may influence host carnitine metabolism or compete for metabolically relevant substrates. *Veillonella* is known to utilize lactate and other host-derived metabolites, and its expansion may divert metabolic resources involved in carnitine synthesis or utilization. Although direct mechanistic evidence is currently lacking, this potential microbe–metabolite interaction warrants further investigation as a contributor to energy dysregulation and inflammation in acute asthma. Although the present study identified the concurrent enrichment of Veillonella and depletion of L-carnitine, a formal microbiome–metabolome integration (e.g., correlation or multivariate integrative analysis) was not performed. This was because microbiome and metabolomic data were not systematically paired across participants, and only a limited number of individuals contributed specimens to both analyses. Importantly, formal integrative modeling on non-paired datasets would be methodologically inappropriate and could lead to spurious associations. Nevertheless, the consistent co-occurrence of these microbial and metabolic alterations supports a plausible microbial–metabolic link that warrants validation in future studies employing fully paired multi-omics designs (Yuan et al., 2022; Wang et al., 2023). These interpretations should therefore be considered hypothesis-generating rather than evidence of direct causality.

Metabolomics pathway enrichment consistently highlighted arginine biosynthesis and metabolism as central and persistently dysregulated pathways (significantly enriched in AA vs. H and CA vs. AA groups). Furthermore, downstream effects of altered arginine metabolism are profound. Reduced arginine bioavailability, potentially exacerbated by dysbiotic microbiota (e.g., shifts in *Streptococcus*/*Prevotella* influencing local nitrogen cycling), can impact nitric oxide (NO) synthesis. Imbalance in NO production, mediated by inducible NOS (iNOS/NOS2) upregulation during inflammation, contributes to airway hyperreactivity, oxidative stress, and epithelial damage–key features reflected in the observed lung function impairments (e.g., reduced FEV1/FVC, FEF25-75%) (Maarsingh et al., 2009).

Furthermore, the strong correlations between environmental metabolites such as dibutyl phthalate/phthalic anhydride and diminished lung function parameters (FVC%pred, FEV1%pred, FEF25-75%pred) underscore the impact of exogenous factors. These pollutants likely induce ferroptosis and oxidative stress, synergizing with microbial and endogenous metabolic dysregulation to worsen airway obstruction and small airway function (Li et al., 2024; Lin et al., 2022). The observed negative correlation between overall microbial abundance (RDA1) and FVC%pred may reflect a loss of beneficial commensals or simple ecological collapse under severe inflammation, contributing to functional decline. Our data suggest that URT microbial dysbiosis (e.g., shifts in dominant taxa like *Streptococcus*/*Prevotella*/*Veillonella*, which can influence local nitrogen cycling and metabolite production) may contribute to this arginine pathway dysregulation, creating a detrimental feedback loop between microbes and host metabolism. These plasticizers are known environmental pollutants linked to asthma development and exacerbation (Mizgerd, 2006; Eales et al., 2022; Gao et al., 2022). Their mechanisms likely involve induction of oxidative stress, promotion of ferroptosis (a form of regulated cell death), and direct pro-inflammatory effects on the airway epithelium and immune cells (Lin et al., 2022). Their presence in the URT metabolome and association with worse lung function underscores the significant contribution of environmental exposures, which may synergize with endogenous microbial and metabolic dysregulation to worsen airway obstruction.

### Limitations

Several limitations of this study should be acknowledged. First, the sample size for metabolomic analysis, particularly in the AA group (*n* = 20), was modest. Importantly, untargeted metabolomics requires substantially higher sample input and metabolite abundance than 16S rRNA gene sequencing, which limits its applicability in low-biomass specimens such as pediatric throat swabs and explains why fewer samples were available for metabolomic analysis compared with microbiome profiling. This imbalance may reduce statistical power and limit the generalizability of metabolomic findings. Second, microbiome and metabolomic data were not systematically paired across participants, which limited the feasibility of formal multi-omics integration analyses. Third, the cross-sectional design precludes causal inference. Longitudinal studies tracking microbiome and metabolome dynamics before, during, and after asthma exacerbations are needed. Finally, throat swabs primarily reflect the oropharyngeal microbiota and may not fully represent the lower airway environment; more invasive sampling approaches could provide deeper insights but are less feasible in pediatric populations.

## Conclusion

1. The abundance and structure of the URT microbiota in children with asthma exhibit specific alterations, with the URT microbiota diversity being lower in those with acute asthma compared to those with chronic persistent asthma.
2. At the genus level, specific URT bacterial genera (*Actinobacillus*, *Fusobacterium*) showed correlations with lung function parameters, including FEV1%/Pred, FEV1/FVC, FEF25%/Pred, FEF50%/Pred, and FEF75%/Pred.
3. The differential metabolite L-carnitine may serve as a potential biomarker for asthma assessment. Metabolic pathways such as arginine biosynthesis, alanine-aspartate–glutamate metabolism, and D-amino acid metabolism may be involved in the pathogenesis of asthma.

## Data Availability

The data presented in this study are publicly available. The data can be found here: https://www.ncbi.nlm.nih.gov, accession number PRJNA1413967.

## Footnotes

1
http://ginasthma.org

## References

[ref1] Cobos-Uribe C. Rebuli M. E. (2023). Understanding the functional role of the microbiome and metabolome in asthma. Curr Allergy Asthma Rep 23, 67–76. doi: 10.1007/s11882-022-01056-9, 36525159 PMC12340730

[ref2] Eales J. Bethel A. Galloway T. Hopkinson P. Morrissey K. Short R. E. . (2022). Human health impacts of exposure to phthalate plasticizers: an overview of reviews. Environ. Int. 158:106903. doi: 10.1016/j.envint.2021.106903, 34601394

[ref3] Galeana-Cadena D. Gómez-García I. A. Lopez-Salinas K. G. Irineo-Moreno V. Jiménez-Juárez F. Tapia-García A. R. . (2023). Winds of change a tale of: asthma and microbiome. Front. Microbiol. 14:1295215. doi: 10.3389/fmicb.2023.1295215, 38146448 PMC10749662

[ref4] Gao D. Zou Z. Li Y. Chen M. Ma Y. Chen L. . (2022). Association between urinary phthalate metabolites and dyslipidemia in children: results from a Chinese cohort study. Environ. Pollut. 295:118632. doi: 10.1016/j.envpol.2021.118632, 34906593

[ref5] Hammad H. Lambrecht B. N. (2021). The basic immunology of asthma. Cell 184, 2521–2522. doi: 10.1016/j.cell.2021.04.019, 33930297

[ref6] Kullberg R. F. J. Haak B. W. Abdel-Aziz M. I. Davids M. Hugenholtz F. Nieuwdorp M. . (2021). Gut microbiota of adults with asthma is broadly similar to non-asthmatics in a large population with varied ethnic origins. Gut Microbes 13:1995279. doi: 10.1080/19490976.2021.1995279, 34743654 PMC8583066

[ref7] Kuruvilla M. E. Lee F. E. Lee G. B. (2019). Understanding asthma phenotypes, endotypes, and mechanisms of disease. Clin. Rev. Allergy Immunol. 56, 219–233. doi: 10.1007/s12016-018-8712-1, 30206782 PMC6411459

[ref8] Li X. Li Z. Ye J. Ye W. (2024). Association between urinary phthalate metabolites and chronic obstructive pulmonary disease: a cross-sectional study. Int. J. Chron. Obstruct. Pulmon. Dis. 19, 1421–1431. doi: 10.2147/COPD.S459435, 38948906 PMC11212814

[ref9] Lin J. Cheng S. Zhang J. Yuan S. Zhang L. Wu J. . (2022). The association between daily dietary intake of riboflavin and lung function impairment related with dibutyl phthalate exposure and the possible mechanism. Nutrients 14:2282. doi: 10.3390/nu14112282, 35684081 PMC9182752

[ref10] Lupu A. Jechel E. Mihai C. M. Mitrofan E. C. Fotea S. Starcea I. M. . (2023). The footprint of microbiome in pediatric asthma-a complex puzzle for a balanced development. Nutrients 15:3278. doi: 10.3390/nu15143278 37513696 PMC10384859

[ref11] Maarsingh H. Zaagsma J. Meurs H. (2009). Arginase: a key enzyme in the pathophysiology of allergic asthma opening novel therapeutic perspectives. Br. J. Pharmacol. 158, 652–664. doi: 10.1111/j.1476-5381.2009.00374.x, 19703164 PMC2765587

[ref12] Man W. H. de Steenhuijsen Piters W. A. Bogaert D. (2017). The microbiota of the respiratory tract: gatekeeper to respiratory health. Nat. Rev. Microbiol. 15, 259–270. doi: 10.1038/nrmicro.2017.14, 28316330 PMC7097736

[ref13] Mashima I. Liao Y. C. Lin C. H. Nakazawa F. Haase E. M. Kiyoura Y. . (2021). Comparative pan-genome analysis of oral *Veillonella* species. Microorganisms 9:1775. doi: 10.3390/microorganisms9081775, 34442854 PMC8400620

[ref14] Mejias A. Ramilo O. (2020). Respiratory syncytial virus treatment and the respiratory microbiome. Lancet Respir. Med. 8, 941–943. doi: 10.1016/S2213-2600(20)30106-5, 32203713

[ref15] Mizgerd J. P. (2006). Lung infection--a public health priority. PLoS Med. 3:e76. doi: 10.1371/journal.pmed.0030076, 16401173 PMC1326257

[ref16] Park H. Shin J. W. Park S. G. Kim W. (2014). Microbial communities in the upper respiratory tract of patients with asthma and chronic obstructive pulmonary disease. PLoS One 9:e109710. doi: 10.1371/journal.pone.0109710, 25329665 PMC4199592

[ref17] Resztak J. A. Choe J. Nirmalan S. Wei J. Bruinsma J. Houpt R. . (2023). Analysis of transcriptional changes in the immune system associated with pubertal development in a longitudinal cohort of children with asthma. Nat. Commun. 14:230. doi: 10.1038/s41467-022-35742-z, 36646693 PMC9842661

[ref18] Rutter C. E. Mpairwe H. Figueiredo C. A. Njoroge M. Robertson S. Ali H. . (2025). Risk factors for atopic and non-atopic asthma in school-age children from high-income and low- and middle-income countries. Thorax 80, 945–954. doi: 10.1136/thorax-2024-222118, 40555500 PMC12703255

[ref19] Scheiman J. Luber J. M. Chavkin T. A. MacDonald T. Tung A. Pham L. D. . (2019). Meta-omics analysis of elite athletes identifies a performance-enhancing microbe that functions via lactate metabolism. Nat. Med. 25, 1104–1109. doi: 10.1038/s41591-019-0485-4, 31235964 PMC7368972

[ref20] Shen S. Wong C. H. (2016). Bugging inflammation: role of the gut microbiota. Clin. Transl. Immunol. 5:e72. doi: 10.1038/cti.2016.12, 27195115 PMC4855262

[ref21] Shumakova A. A. Shipelin V. A. Leontyeva E. V. Gmoshinski I. V. (2022). Effect of resveratrol, L-carnitine, and aromatic amino acid supplements on the trace element content in the organs of mice with dietary-induced obesity. Biol. Trace Elem. Res. 200, 281–297. doi: 10.1007/s12011-021-02642-0, 33624220

[ref22] Takai K. Horikoshi K. (2000). Rapid detection and quantification of members of the archaeal community by quantitative PCR using fluorogenic probes. Appl. Environ. Microbiol. 66, 5066–5072. doi: 10.1128/AEM.66.11.5066-5072.2000, 11055964 PMC92420

[ref23] Valverde-Molina J. García-Marcos L. (2023). Microbiome and asthma: microbial dysbiosis and the origins, phenotypes, persistence, and severity of asthma. Nutrients 15:486. doi: 10.3390/nu15030486, 36771193 PMC9921812

[ref24] Walters W. Hyde E. R. Berg-Lyons D. Ackermann G. Humphrey G. Parada A. . (2016). Improved bacterial 16S rRNA gene (V4 and V4-5) and fungal internal transcribed spacer marker gene primers for microbial community surveys. mSystems 1:e00009-15. doi: 10.1128/mSystems.00009-15, 27822518 PMC5069754

[ref25] Wang W. Dernst A. Martin B. Lorenzi L. Cadefau-Fabregat M. Phulphagar K. . (2024). Butyrate and propionate are microbial danger signals that activate the NLRP3 inflammasome in human macrophages upon TLR stimulation. Cell Rep. 43:114736. doi: 10.1016/j.celrep.2024.114736, 39277863

[ref26] Wang C. Yu X. Lin H. Wang G. Liu J. Gao C. . (2023). Integrating microbiome and metabolome revealed microbe-metabolism interactions in the stomach of patients with different severity of peptic ulcer disease. Front. Immunol. 14:1134369. doi: 10.3389/fimmu.2023.1134369 36969184 PMC10034094

[ref27] Yang C. H. Li X. Y. Lv J. J. Hou M. J. Zhang R. H. Guo H. . (2024). Temporal trends of asthma among children in the Western Pacific region from 1990 to 2045: longitudinal observational study. JMIR Public Health Surveill. 10:e55327. doi: 10.2196/55327, 38483459 PMC10979332

[ref28] Yuan Y. Wang C. Wang G. Guo X. Jiang S. Zuo X. . (2022). Airway microbiome and serum metabolomics analysis identify differential candidate biomarkers in allergic rhinitis. Front. Immunol. 12:771136. doi: 10.3389/fimmu.2021.771136 35069544 PMC8766840

[ref29] Zhang M. Qin Z. Huang C. Liang B. Zhang X. Sun W. (2025). The gut microbiota modulates airway inflammation in allergic asthma through the gut-lung axis related immune modulation: a review. Biomol. Biomed. 25, 727–738. doi: 10.17305/bb.2024.11280, 39465678 PMC11959394

[ref30] Zheng P. Zhang K. Lv X. Liu C. Wang Q. Bai X. (2022). Gut microbiome and metabolomics profiles of allergic and non-allergic childhood asthma. J. Asthma Allergy 15, 419–435. doi: 10.2147/JAA.S354870, 35418758 PMC8995180

