# Supplementary figures and images for: Microbial and metabolomic profiling of the upper respiratory tract in children with asthma
Source: Front Microbiol. 2026 Feb 17;17:1672589. doi: 10.3389/fmicb.2026.1672589 (PMC12954610; doi:10.3389/fmicb.2026.1672589)

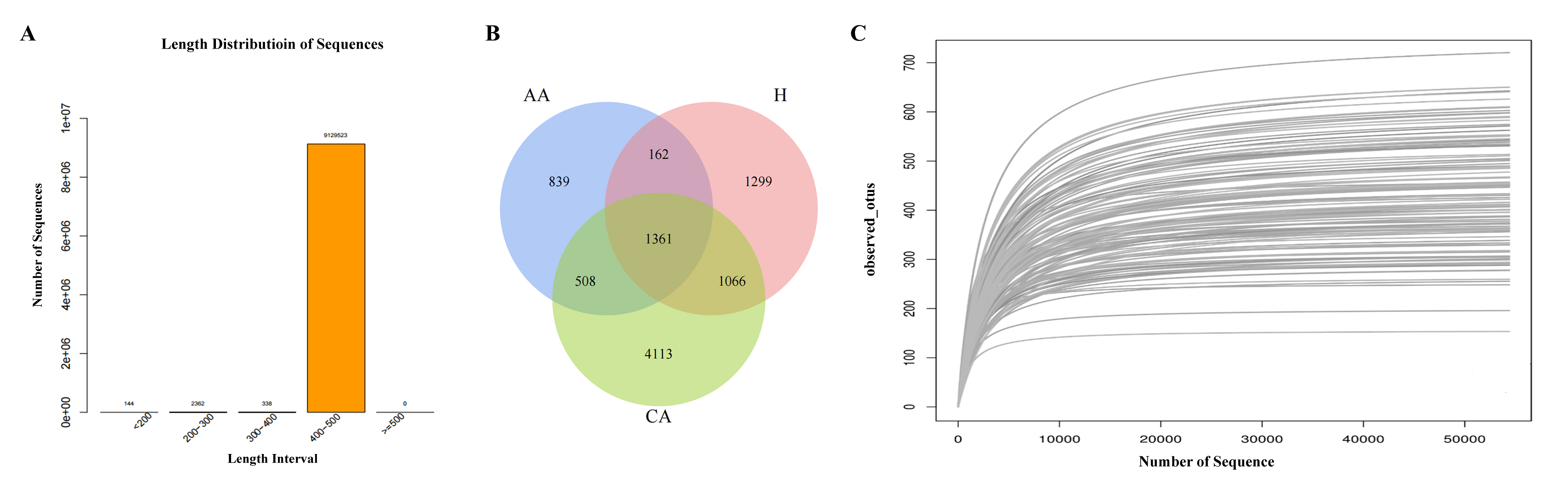

Supplement: Supplementary file 1 [file Image_1.tif]

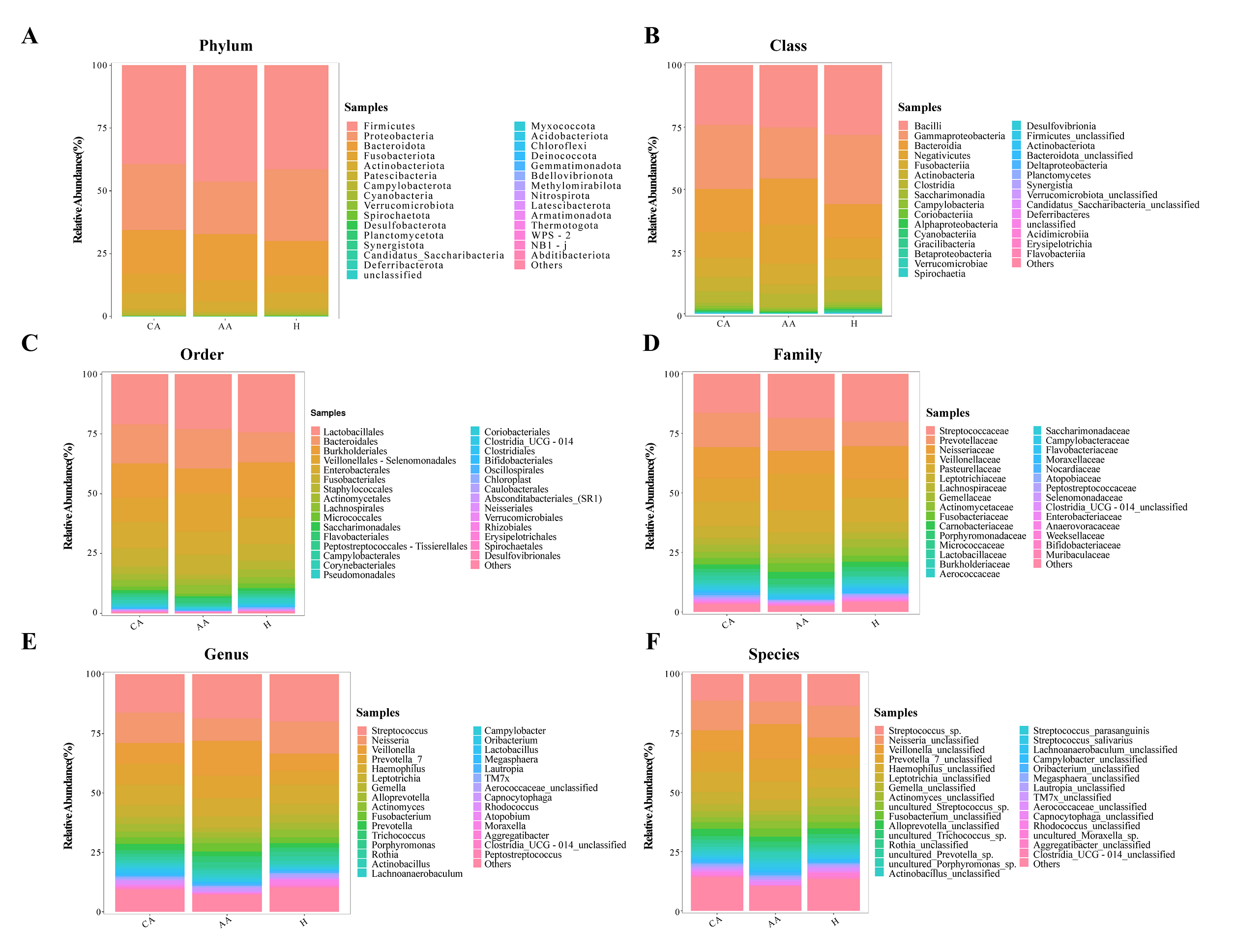

Supplement: Supplementary file 2 [file Image_2.tif]
