# Supplementary material for: Microbial and metabolomic profiling of the upper respiratory tract in children with asthma
Source: Front Microbiol. 2026 Feb 17;17:1672589. doi: 10.3389/fmicb.2026.1672589 (PMC12954610; doi:10.3389/fmicb.2026.1672589)
Supplement: Supplementary file 3 [file Supplementary_file_1.docx]

**Supplementary materials Table S1：Clinical characteristics analysis** **of CA AA and H group**

| Characteristics | CA | AA | H | *P* |
| --- | --- | --- | --- | --- |
| Number of cases | 78 | 21 | 27 | - |
| Sex(male/female) | 50/28 | 11/10 | 16/11 | 0.607 |
| Age（year） | 8.5（7,10） | 10(7,11) | 9(8,10) | 0.626 |
| BMI（kg/m^2^） | 17.3（15.4，20.1） | 16.3(14.5,22.4) | 16.8(15.7,17.6) | 0.483 |

**Table S2： Analysis of lung function test indicators during CA and AA group**

| Characteristics | CA | AA | *P* |
| --- | --- | --- | --- |
| FVC%/Pred | 101.6(93.2,108.2) | 91.2(73.3,100.1) | ＜0.01 |
| FEV1%/Pred | 96.8(88.7,106.3) | 80.4(56,91.5) | ＜0.01 |
| FEV1/FVC | 96.3(91.1,100.7) | 88.1(78.2,94.3) | ＜0.01 |
| PEF%/Pred | 90.6(81.9,101.5) | 70.3(51.9,84.2) | ＜0.01 |
| FEF25%/Pred | 90.0(77.3,103.7) | 71.4(44.3,82.6) | ＜0.01 |
| FEF50%/Pred | 76.6(65.1,89.9) | 61.8(26.7,73.3) | ＜0.01 |
| FEF75%/Pred | 59.5(48.7,73.3) | 39.1(23.8,62.7) | ＜0.01 |

**Table S3：Clinical characteristics analysis of CA AA and H group**

|  | | CA | AA | H | *P* |
| --- | --- | --- | --- | --- | --- |
| Number of cases | 28 | | 20 | 27 | - |
| Sex(male/female) | 16/12 | | 11/9 | 16/11 | 0.959 |
| Age（year） | 10（7,12） | | 9（8,10） | 9（8,10） | 0.346 |
| BMI  （kg/m^2^） | 16.5(15.0,22.9) | | 16.4(15.1,18.2） | 16.8(15.7,17.6) | 0.765 |
| Living environment (urban/rural) | 21/7 | | 18/2 | 23/4 | 0.369 |

**Table S4： Comparison of lung function parameters between CA and AA group**

|  | AA | CA | *P* |
| --- | --- | --- | --- |
| FVC%/Pred | 94.2（78.2,100.2） | 98.3（88.8,107.1） | 0.027* |
| FEV1%/Pred | 84.6（60.3,95.5） | 95.7（86.9,101.8） | ＜0.01 |
| FEV1/FVC | 91.7（78.2,100.2） | 98.3（93.1,101.6） | ＜0.01 |
| PEF%/Pred | 79.8（54.6,88.1） | 92.7（82.1,101.1） | ＜0.01 |
| FEF25%/Pred | 76.2（59.2,82.8） | 89.8（79.2,97.9） | ＜0.01 |
| FEF50%/Pred | 65.2（32.7,75.9） | 78.7（70.3,90.4） | ＜0.01 |
| FEF75%/Pred | 50.0（34.4,65.3） | 61.1（44.8,74.1） | 0.083 |

**Table S5. Baseline characteristics of the overall microbiome cohort and the metabolomics subset**

| Characteristics | Overall microbiome cohort (n = 126 ) | Metabolomics subset (n = 75) | *P* |
| --- | --- | --- | --- |
| Sex(male/female) | 77/49 | 43/32 | 0.502 |
| Age（year） | 9.5（7,10） | 10(7,11) | 0.635 |
| BMI（kg/m^2^） | 16.1（15.1，20.5） | 16.3(14.5,22.4) | 0.483 |
| Lung function (AA) |  |  |  |
| FVC%/Pred | 91.2(73.3,100.1) | 94.2（78.2,100.2） | 0.535 |
| FEV1%/Pred | 80.4(56,91.5) | 84.6（60.3,95.5） | 0.231 |
| FEV1/FVC | 88.1(78.2,94.3) | 91.7（78.2,100.2） | 0.421 |
| PEF%/Pred | 70.3(51.9,84.2) | 79.8（54.6,88.1） | 0.145 |
| Lung function (CA) |  |  |  |
| FVC%/Pred | 101.6(93.2,108.2) | 98.3（88.8,107.1） | 0.383 |
| FEV1%/Pred | 96.8(88.7,106.3) | 95.7（86.9,101.8） | 0.781 |
| FEV1/FVC | 96.3(91.1,100.7) | 98.3（93.1,101.6） | 0.432 |
| PEF%/Pred | 90.6(81.9,101.5) | 92.7（82.1,101.1） | 0.546 |

**Table S6** 9 common differential metabolite names and SuperClass classification in HMDB for each group

| Metabolite name | SuperClass |
| --- | --- |
| Crotamiton | Benzenoids |
| Phosphocholine | Organic nitrogen compounds |
| all-trans-Retinoic acid methyl ester | - |
| dehydro-beta-Ionone | Lipids and lipid-like molecules |
| 1,2-Dihydro-1,1,6-trimethylnaphthalene | Benzenoids |
| 2-trans-6-cis-Dodecadienal | Organic oxygen compounds |
| 2-Hexyl-4-pentynoic acid | - |
| Tebuconazole  Eremopetasinorol | -  Organooxygen compounds |

**Table S7** Differential metabolite pathway results between H and AA group

| Pathway_Name | Rich Factor | *P* | | -log_10_(*P*) | Up_Num | Down_Num |
| --- | --- | --- | --- | --- | --- | --- |
| Arginine biosynthesis | 0.26 | | 3.1E-9 | 8.50 | 0 | 6 |
| Alanine, aspartate and glutamate  metabolism | 0.21 | | 1.1E-8 | 7.94 | 0 | 6 |
| Central carbon metabolism in cancer | 0.16 | | 6.7E-8 | 7.17 | 1 | 5 |
| D-Amino acid metabolism | 0.11 | | 1.4E-7 | 6.85 | 1 | 6 |
| Protein digestion and absorption | 0.13 | | 3.0E-7 | 6.52 | 1 | 5 |
| Aminoacyl-tRNA biosynthesis | 0.12 | | 5.5E-7 | 6.25 | 1 | 5 |
| Neuroactive ligand-receptor interaction | 0.11 | | 6.2E-7 | 6.20 | 2 | 4 |

**Table S8** Differential metabolite pathway results between CA and AA group

| Pathway_Name | Rich Factor | *P* | -log_10_(*P*) | Up_Num | Down_Num | | |
| --- | --- | --- | --- | --- | --- | --- | --- |
| Arginine biosynthesis | 0.30 | 2.6E-11 | 10.57 | 0 | | 7 |  |
| Alanine, aspartate and  glutamate metabolism | 0.25 | 1.2E-10 | 9.90 | 0 | | 7 |  |
| Central carbon metabolism in cancer | 0.19 | 1.1E-9 | 8.98 | 1 | | 6 |  |
| D-Amino acid metabolism | 0.12 | 3.5E-9 | 8.45 | 1 | | 7 |  |

Rich Factor: The higher the value, the higher the degree of enrichment. Up-Num: The number of significantly upregulated metabolites, Down-Num: The number of significantly downregulated metabolites.

**Supplementary materials Figure S1：Quality Assessment and Diversity Analysis of Respiratory Microbiome Sequencing Data** (A) Sequence Length Distribution. (B). Amplicon Sequence Variants (ASVs) Distribution Venn Diagram. (C). Rarefaction Curve

A total of 126 samples were analyzed, yielding 10,594,426 raw sequences. After quality control, 9,132,367 high-quality sequences were retained (retention rate: 86%). The majority of sequences (99.9%) were concentrated in the 400–500 bp range, meeting the requirements for downstream analyses(Figure S1A). A total of 9,348 Amplicon Sequence Variants (ASVs) were identified through 16S rDNA sequencing. The Venn diagram illustrates shared and unique ASVs across clinical groups (e.g., chronic asthma vs. acute exacerbation), highlighting conserved microbial features among subgroups(Figure S1B).Rarefaction curves for all samples plateaued, indicating that the sequencing depth adequately captured microbial diversity. This validates the sufficiency of data volume for subsequent α/β diversity and taxonomic analyses(Figure S1C).

**Figure S2：Species classification bar chart**

At the phylum level, the control group was mainly composed of Firmicutes (41.34%), Proteobacteria (28.61%), Bacteroidota (13.71%), Fusobacteriota (7.01%), and Actinobacteriota (6.25%). The chronic persistent asthma group was mainly composed of Firmicutes (39.30%), Proteobacteria (26.33%), Bacteroidota (17.31%), Fusobacteriota (7.83%), and Actinobacteriota (6.40%). The acute exacerbation group was mainly composed of Firmicutes (46.33%), Proteobacteria (20.92%), Bacteroidota (18.45%), Fusobacteriota (8.31%), and Actinobacteriota (4.19%) (Figure S2A).

At the class level, the control group was mainly composed of Bacilli (28.08%), Gammaproteobacteria (27.81%), Bacteroidia (13.69%), and Negativicutes (8.40%). The chronic persistent asthma group was mainly composed of Bacilli (24.17%), Gammaproteobacteria (25.68%), Bacteroidia (17.29%), and Negativicutes (10.34%). The acute exacerbation group was mainly composed of Bacilli (25.11%), Gammaproteobacteria (20.57%), Bacteroidia (28.45%), and Negativicutes (15.79%) (Figure S2B).

At the order level, the control group was mainly composed of Lactobacillales (24.37%), Burkholderiales (14.62%), Bacteroidales (12.53%), and Enterobacterales (11.15%). The chronic persistent asthma group was mainly composed of Lactobacillales (20.94%), Burkholderiales (16.41%), Bacteroidales (14.24%), and Enterobacterales (10.78%). The acute exacerbation group was mainly composed of Lactobacillales (22.94%), Bacteroidales (16.51%), Veillonellales-Selenomonadales (15.75%), and Burkholderiales (10.55%) (Figure S2C).

At the family level, the control group was mainly composed of Streptococcaceae (20.11%), Neisseriaceae (13.82%), Prevotellaceae (10.25%), and Pasteurellaceae (10.22%). The chronic persistent asthma group was mainly composed of Streptococcaceae (16.31%), Prevotellaceae (14.49%), Neisseriaceae (12.99%), and Pasteurellaceae (10.23%). The acute exacerbation group was mainly composed of Streptococcaceae (18.59%), Veillonellaceae (15.48%), Prevotellaceae (13.79%), and Neisseriaceae (9.52%) (Figure S2D).

At the genus level, the control group was mainly composed of Streptococcus (20.02%), Neisseria (13.38%), Haemophilus (8.16%), and Veillonella (7.31%). The chronic persistent asthma group was mainly composed of Streptococcus (20.02%), Neisseria (12.68%), Veillonella (8.84%), and Prevotella (8.71%). The acute exacerbation group was mainly composed of Streptococcus (18.54%), Veillonella (14.74%), Prevotella (9.49%), and Neisseria (9.45%) (Figure S2E).

At the species level, the control group was mainly composed of Streptococcus species (13.42%), unclassified Neisseria species (13.36%), unclassified Haemophilus species (8.16%), and unclassified Veillonella species (7.31%). The chronic persistent asthma group was mainly composed of unclassified Neisseria species (12.67%), Streptococcus species (11.16%), unclassified Veillonella species (8.84%), and unclassified Prevotella species (8.71%). The acute exacerbation group was mainly composed of unclassified Veillonella species (14.74%), Streptococcus species (11.66%), unclassified Prevotella species (9.49%), and unclassified Neisseria species (9.44%) (Figure S2F).
